# Essential role of microglia in the fast antidepressant action of ketamine and hypidone hydrochloride (YL-0919)

**DOI:** 10.3389/fphar.2023.1122541

**Published:** 2023-05-26

**Authors:** Hai-Xia Chang, Wei Dai, Jin-Hao Bao, Jin-Feng Li, Ji-Guo Zhang, Yun-Feng Li

**Affiliations:** ^1^ College of Pharmacy, Shandong First Medical University and Shandong Academy of Medical Sciences, Taian, China; ^2^ Beijing Institute of Basic Medical Sciences, Beijing, China; ^3^ State Key Laboratory of Toxicology and Medical Countermeasures, Beijing Key Laboratories of Neuropsychopharmacology, Institute of Pharmacology and Toxicology, Beijing, China

**Keywords:** microglia, fast antidepressant action, ketamine, YL-0919, synaptic proteins

## Abstract

**Introduction:** Intracerebral microglia play a vital role in mediating central immune response, neuronal repair and synaptic pruning, but its precise role and mechanism in fast action of antidepressants have remained unknown. In this study, we identified that the microglia contributed to the rapid action of antidepressants ketamine and YL-0919.

**Methods:** The depletion of microglia was achieved with the diet containing the colony-stimulating factor 1 receptor (CSF1R) inhibitor PLX5622 in mice. The tail suspension test (TST), forced swimming test (FST) and novelty suppressed feeding test (NSFT) were employed to evaluate the rapid acting antidepressant behavior of ketamine and YL-0919 in the microglia depletion model. The number of microglia in the prefrontal cortex (PFC) was assayed by the immunofluorescence staining. The expressions of synaptic proteins (synapsin-1, PSD-95, GluA1) and brain-derived neurotrophic factor (BDNF) in the PFC were tested by Western blot.

**Results:** The immobility duration in FST and the latency to feed in NSFT were shortened 24 h after an intraperitoneal (i.p.) injection of ketamine (10 mg/kg). The microglial depletion of PLX3397 blocked the rapid antidepressant-like effect of ketamine in mice. In addition, the immobility time in TST and FST as well as latency to feed in NSFT were reduced 24 h after the intragastric (i.g.) administration of YL-0919 (2.5 mg/kg, administered for 5–6 consecutive days), and the rapid antidepressant effect of YL-0919 was also blocked by the microglial depletion using PLX5622. About 92% of microglia in the prefrontal cortex was depleted in PLX5622 diet-fed mice, while both ketamine and YL-0919 promoted proliferation on the remaining microglia. YL-0919 significantly increased the protein expressions of synapsin-1, PSD-95, GluA1 and BDNF in the PFC, all of which could be blocked by PLX5622.

**Conclusion:** These results suggested the microglia underlying the rapid antidepressant-like effect of ketamine and YL-0919, and microglia would likely constitute in the rapid enhancing impact of synaptic plasticity in the prefrontal cortex by YL-0919.

## Introduction

Depression, as an affective disorder, is characterized mainly by persistent and recurrent dysthymia, and has become a serious global public health challenge. The pathogenesis of depression is still unclear. The clinical antidepressants are mainly based on “monoamine strategy,” such as selective serotonin reuptake inhibitors (SSRIs) and serotonin and norepinephrine reuptake inhibitors (SNRIs). Most of first-line antidepressants have defects such as delayed onset of action, limited efficacy (50%–70%), cognition impairment, sexual dysfunction and possible suicidal tendency (Delgado, 2000; Li, 2020). The new antidepressant mechanism is of great significance to the development of fast-acting and cognition-enhancing new drugs.

As the resident immune cell in the central nervous system (CNS), microglia was related to the pathogenesis of depression (Hodes et al., 2015; Miller and Raison, 2016). The levels of pro-inflammatory cytokines (such as IL-6, TNF-α) were significantly increased in the serum of patients with depression (Buglione-Corbett et al., 2018). The injection of lipopolysaccharide (LPS, i.p.) could cause depressive-like behaviors with elevated cerebral inflammatory response and microglia activation (Zhao et al., 2019). Other studies suggested that the inflammatory response was associated with the risk of depression (Setiawan et al., 2015). By clearance of pathogens and toxic cell debris, reactive microglia released a variety of inflammatory cytokines (IL-1β,IL-6,TNF-α), which caused chronic neuroinflammation and participated in the progression of depression (Jia et al., 2021). Regulation of microglia could be the potential strategy for depression treatment (Farhangian et al., 2023). Nevertheless, the role and mechanism of microglia in rapid action of antidepressants were far from elucidation.

Fluoxetine, a traditional SSRI antidepressant, significantly reduced LPS-induced activation of primary microglia and release of inflammatory cytokines IL-6, TNF-α by inhibiting NF-κB (Yang et al., 2014). Ketamine was reported as a new rapid acting antidepressant marketed in the United States in 2019 (*s*-ketamine), and extensive clinical evidences demonstrated that a single subanesthetic dose of ketamine produced antidepressant efficacy lasting for 1 week (Zanos and Gould, 2018; Corriger and Pickering, 2019; Lima et al., 2022). The preliminary and limited study indicated that the depletion of cortical microglia could block the antidepressant effect of (*R*)-ketamine on chronic social defeat stress (CSDS) susceptible mice, suggesting that microglia might be involved in the antidepressant effect of (*R*)-ketamine (Yao et al., 2022).

YL-0919, a novel antidepressant compound developed by our institute, was tested to be a sigma-1 receptor agonist (Ren et al., 2023). YL-0919 exerted a rapid onset of antidepressant action in multiple animal models, and had the advantages of cognition enhancing effect and not causing sexual dysfunction (Chen et al., 2018; Ran et al., 2018; Sun et al., 2019; Yin et al., 2020). The long-term potentiation (LTP) recording showed that YL-0919 significantly enhanced the hippocampal neuroplasticity of rats within 7d (while 21d for fluoxetine) (Zhang et al., 2017). Further multichannel electrophysiological technique suggested that YL-0919 preferentially inhibited GABAergic interneurons in the PFC and enhanced excitability of Glutamatergic pyramidal neurons. YL-0919 then rapidly regulated excitation/inhibition (E/I) balance in the PFC, and upregulated BDNF-mammalian target of rapamycin (mTOR) pathway to improve neural plasticity (Zhang et al., 2022), which was consistent with the rapid acting antidepressant mechanism of ketamine (Homayoun and Moghaddam, 2007; Autry et al., 2011; Moda-Sava et al., 2019). The adjustment of synaptic plasticity and E/I balance in the PFC might be a critical part of rapid action of antidepressants (Li, 2020). However, those related studies have largely focused on neurons, and the implications of microglia on rapid acting antidepressant effect are still unknown.

This study aims to discover the microglial mechanism in rapid action of antidepressants (ketamine as a control). The effect of microglial depletion on the rapid action of ketamine and YL-0919 was identified by CSF1R inhibitor PLX5622 in mice. The microglial depletion ratio of PLX5622 and the effect of ketamine/YL-0919 on the number of microglia were assessed by immunofluorescence in the PFC. At last, the impact of microglial depletion on the YL-0919-induced upregulated synaptic proteins and BDNF were observed to demonstrate a new antidepressant mechanism of YL-0919.

## Material and methods

### Animals

Male adult C57BL/6J mice, aged 8 weeks (21–23 g) were purchased from SPF (Beijing) Biotechnology. The mice were housed in groups of 5 per cage at constant temperature (22°C ± 2°C), in a humid environment (55% ± 10%) and exposed to a light/dark cycle of 12 h for 7d of acclimatization. The mice were provided with food and water freely. All procedures were performed in compliance with the National Institutes of Health Guide for the Care and Use of Laboratory Animals, and approved by the institutional committee on animal care and use.

### Drugs and treatment

YL-0919 was provided by the Institute of Pharmacology and Toxicology (with purity>99.9%). Ketamine was purchased from Fort Dodge Animal Health (Fort Dodge, IA, United States). Ketamine and YL-0919 was dissolved in distilled water and administered respectively at a volume of 10 mL/kg. The depletion of microglia was achieved by the CSF1R inhibitor PLX5622 for 19 days (1200 PPM added to chow AIN-76A, SYSEBIO). On the sixteenth day of PLX5622 or vehicle chow (AIN-76A) treatments, ketamine (10 mg/kg, i.p.) was administered intraperitoneally 24 h prior to behavioral tests. Mice were divided into 4 groups, and each group contained 9-10 mice.

**FIGURE 1:**
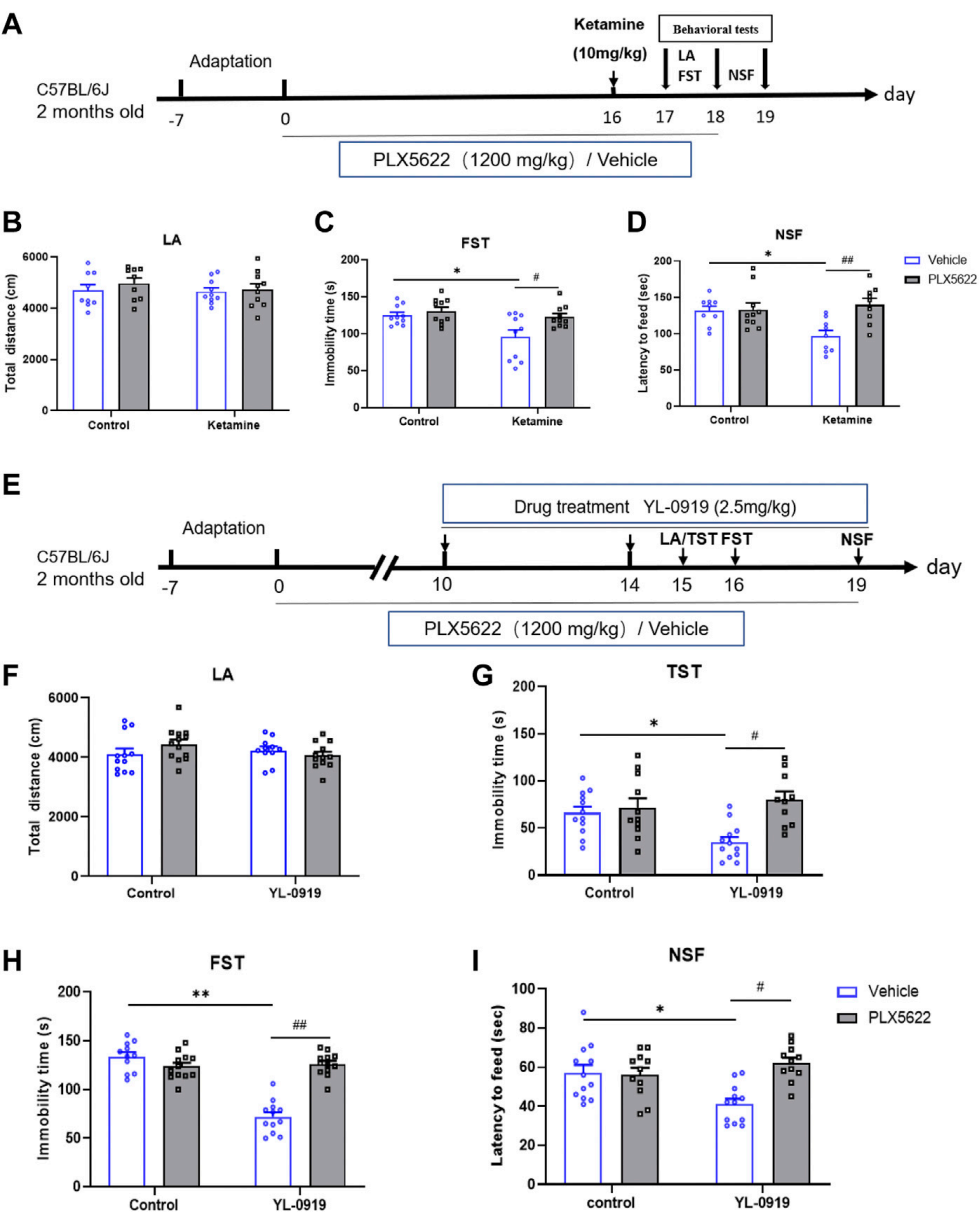
Schematic diagram illustrating the effects of PLX5622 on rapid antidepressant action of ketamine **(A)**; Effects of PLX5622 on behavioral tests including locomotor activity **(B)**, immobility duration in FST **(C)** and latency to feed **(D)** in ketamine-treated mice. veh + control vs. veh + ketamine, *p < 0.05; veh + ketamine vs. PLX5622+ketamine, ^#^ *p* < 0.05, ^##^ *p* < 0.01, *n* = 9-10. Schematic diagram illustrating the effects of PLX5622 on rapid antidepressant action of YL-0919 **(E)**; Effects of PLX5622 on behavioral tests including locomotor activity **(F)**, immobility duration in TST **(G)** and FST **(H)**; and latency to feed **(I)** in YL-0919-treated mice, veh + control vs. veh + YL-0919, *p < 0.05, **p < 0.01; veh + YL-0919 vs. PLX5622+YL-0919, ^#^ *p* < 0.05, ^##^ *p* < 0.01; Means ± SEM, *n* = 11–12.

On the tenth day of PLX5622 or vehicle chow (AIN-76A) treatments, YL-0919 (2.5 mg/kg, i.g.) was administered twice (8:00 a.m. and 8:00 p.m.) for 5 consecutive days, and was then treated once (8:00 p.m.) during the testing period. Behavioral tests were performed separately 24 h after the administration of YL-0919. Mice were divided into 4 groups, and each group contained 11-12 mice. The procedures of drug treatments and tests were demonstrated in Figures 1A, E.

### Locomotor activity test (LA)

The LA was performed following previously described procedures (Liu et al., 2021; Zhang et al., 2014). The mice were placed into the test cages (with 16 grids divided equally at the bottom), and the head of each mouse was directed towards a fixed corner of the cage. The mice were allowed to explore the apparatus freely for 5 min. The SMART video software was adopted to record and analyze the locomotor activity of mice. Cages were cleaned between testing session.

### Tail suspension test (TST)

The TST was performed following previously described procedures (Castagné et al., 2011). The Medical adhesive tape was used to fix the tail of each mouse (approximately 1.5 cm from the tip of the tail for mouse) on the top of apparatus. The behaviors of the mice were recorded with the camera for 6 min. The cumulative immobility duration of each mouse for the last 4 min was analyzed.

### Forced swimming test (FST)

The FST was performed following previously described procedures (Castagné et al., 2011). The mice were placed into an open glass apparatus (height: 20 cm, diameter: 14 cm) filled with 12 cm of water (23°C ± 1°C). The mice were forced to swim freely and recorded with the camera for 6 min. The cumulative immobility duration of the mice for the last 4 min was measured.

### Novelty suppressed feeding test (NSFT)

The NSF was performed following previously described procedures (Liu et al., 2021). The mice and they were deprived of food for 24 h (access to unlimited water) and adapt to a quiet environment for 60 min before the experiment. Each mouse was placed in one corner of an open test box (covered with 1 cm clean bedding) with 5 pellets in the center. The latency to the feed within 5 min was recorded, and the feeding behavior was defined as chewing and biting by mice.

### Immunofluorescence

The mice were anesthetized with 1% pentobarbital sodium and perfused with 4% paraformaldehyde (PFA). Brains were extracted and fixed in 4% PFA. The tissues were stored in different sucrose solutions for gradient dehydration and then sectioned with a cryostat at 30 μm. The sections were washed with PBS buffer (pH = 7.4) for 3 times and blocked with PBS containing 0.3% Triton-X and 10% normal goat serum at room temperature for 2 h. Sections were then incubated with primary antibody of Iba1 (1:200, abcam, ab178846) overnight at 4°C, Following washes in PBS, the tissues were incubation with biotinylated goat anti-rabbit IgG secondary antibody (1:2000, CST, #4412) for 2 h at room temperature in the dark. After washing, the sections were mounted and cover-slipped with anti-fluorescence quenching agent containing DAPI on slides. Immunofluorescence imaging was examined using confocal microscopy (Nikon A1). ImageJ was measured to analyze the number of microglia.

### Western blotting

The mice were decapitated for sacrifice, and the prefrontal cortex was dissected and stored at −80°C. Cortical tissues were homogenized, and centrifuged at 12,000×g for 10 min. The supernatant was recovered, and protein concentration was evaluated using BCA method. Samples containing 30 μg of proteins were loaded onto the SDS polyacrylamide gel and transferred to PVDF membranes. The membrane was blocked with 5% skim milk, and incubated separately with primary antibodies of Iba1(1:1,000, abcam, ab178846), synapsin-1 (1:1,000, CST, #5297), GluA1 (1:1,000, CST, #8084), PSD-95 (1:1,000, CST, #3450), BDNF (1:1,000, abcam, ab108319), sigma-1 (1:1,000, Santacruz, #sc137075) or β-actin (1:1,000, Cwbio) overnight at 4°C. The membrane was washed 3 times with TBST, and incubated in fluoresce-labeled secondary antibodies for 2 h at room temperature in the dark. Images was visualized using an Odyssey two-color infrared imaging system.

### Statistical analysis

The statistical data are represented as Means ± SEM. In the behavior tests, experiments were performed blind. Results were analyzed using Student’s t-test or the two-way ANOVA followed by Tukey’s multiple comparisons test. The *p* < 0.05 was considered statistically significant.

## Results

### Microglial depletion restrained the rapid antidepressant action of ketamine and YL-0919

To investigate whether microglia contribute to fast action of ketamine and YL-0919, depletion of microglia in mice was achieved with the diet containing PLX5622 (Figures 1A, E). There were no changes in locomotion activity among the different groups (Figures 1B, F, *p* > 0.05). Compared with the control group, the immobility durations in FST and NSFT were significantly decreased by ketamine (10 mg/kg, i. p.) injection, indicating a rapid acting antidepressant effect of ketamine [F (1, 36) = 6.742, *p* = 0.0135; F (1, 33) = 7.807, *p* = 0.0036; Figures 1C, D, *p* < 0.05]. In addition, the immobility durations of mice in FST and TST were significantly reduced by the administration of YL-0919 (2.5 mg/kg, i.g., administered for 5-6 consecutive days) [F (1, 41) = 10.62, *p* = 0.0279; F (1, 50) = 20.15, *p* = 0.0001; Figures 1G, H, *p* < 0.05]. The latency to feed was also reduced by YL-0919 (administered for 9 consecutive days) [F (1, 43) = 10.19, *p* = 0.0142; Figure 1I, *p* < 0.05]. YL-0919 preformed a rapid acting antidepressant effect, which was consistent with the onset time of action from previous studies in our lab (Chen et al., 2018; Ran et al., 2018; Sun et al., 2019). Interestingly, compared with ketamine-treated group, depletion of microglia restrained the fast antidepressant action of ketamine for increased immobility durations of FST and latency to feed [F (1, 36) = 8.283, *p* = 0.0223; F (1, 33) = 2.996, *p* = 0.0246; Figures 1C, D, *p* < 0.05]. Compared to YL-0919 group, microglial depletion by PLX5622 also significantly blocked the fast antidepressant action of YL-0919 for increased immobility durations of TST, FST and latency to feed [F (1, 41) = 2.115, *p* = 0.0012; F (1, 50) = 44.88, *p* = 0.0001; F (1, 43) = 1.688, *p* = 0.0004; Figures 1G–I, *p* < 0.05]. However, PLX5622 itself had no impact on the above behaviors. These results suggested that microglia played an essential role in fast antidepressant action of ketamine and YL-0919.

### Effect of PLX5622 on the number of microglia in the PFC of ketamine and YL-0919-treated mice

**FIGURE 2:**
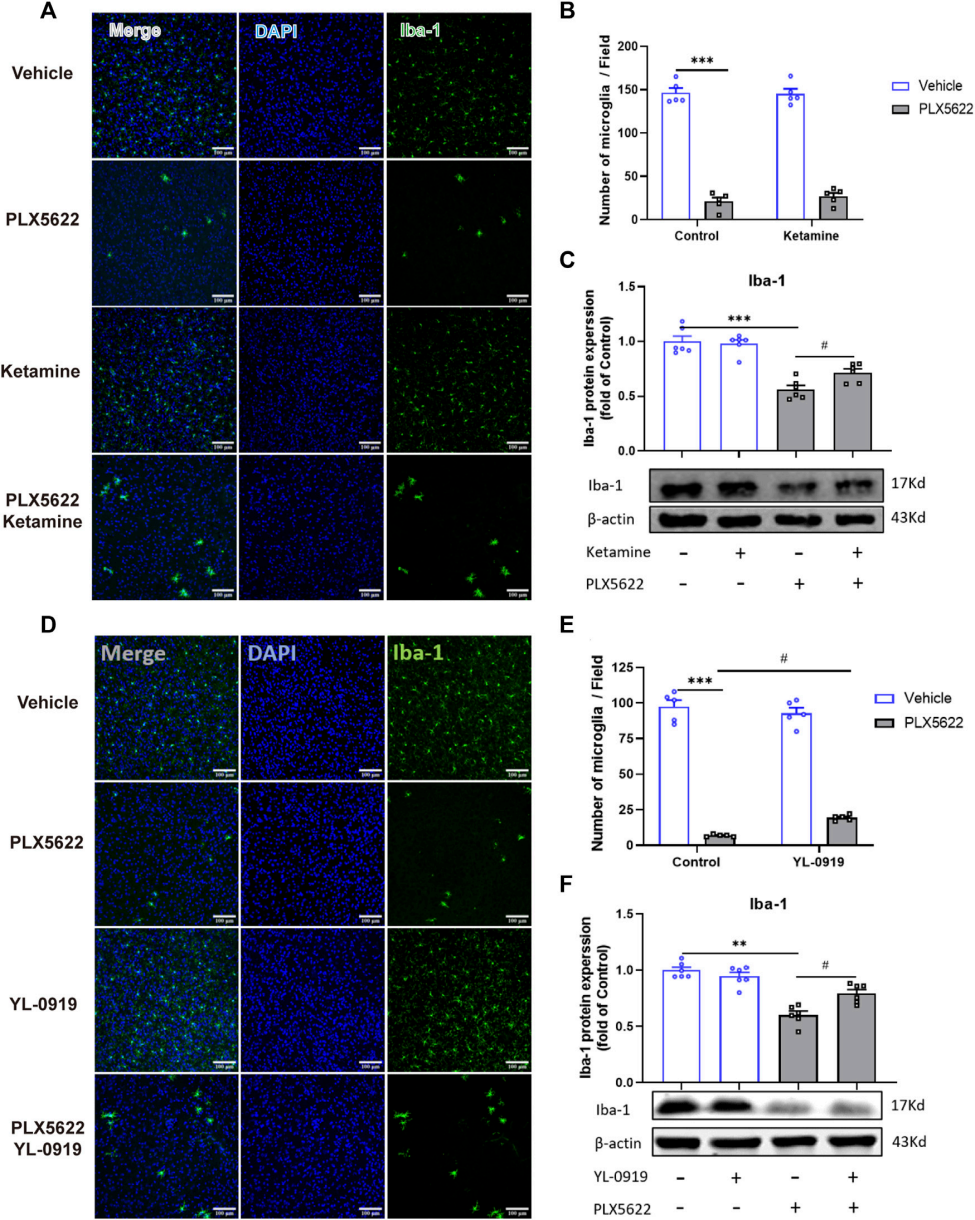
Effects of PLX5622 on the density of microglia in the PFC of ketamine and YL-0919-treated Mice. Representative immunofluorescence image and analysis of Iba1(Green, marker for microglia) in the PFC of PLX5622 and ketamine-treated mice **(A, B)**, the Iba1 protein expression in the PFC of PLX5622 and ketamine-treated mice **(C)**, veh + control vs. PLX5622+ control, ***p < 0.001, PLX5622+ control vs PLX5622+ ketamine, ^#^ *p* < 0.05; Representative immunofluorescence image and analysis of Iba1 in the PFC of PLX5622 and YL-0919-treated mice **(D, E)**, the Iba1 protein expression in the PFC of PLX5622 and YL-0919-treated mice **(F)**. scale bar = 100 μM;veh + control vs. PLX5622+ control, **p < 0.01, ***p < 0.001; PLX5622+ control vs. PLX5622+YL-0919, ^#^ *p* < 0.05, Means ± SEM, *n* = 5.

The effect of PLX5622 on microglial density and the regulation of ketamine and YL-0919 on the number of microglia were validated by immunofluorescence staining in the PFC (Figures 2A, D). Compared with the control group, ketamine and YL-0919 administration did not affect the number of microglia in the PFC (Figures 2B, C, E, F, *p* > 0.05). After PLX5622 diet, the density of microglia in the PFC was reduced by 92% [F (1, 16) = 0.2320, *p* = 0.0001; F (1, 16) = 1.716, *p* = 0.0001; Figures 2B, E, *p* < 0.05], and the expression of Iba1 protein in the PFC was significantly decreased (t = 7.098, *p* = 0.0001; t = 8.929, *p* = 0.0001; Figures 2C, F, *p* < 0.05). However, both ketamine and YL-0919 promoted the proliferation of the remaining microglia in the PFC of mice treated with PLX5622 (t = 3.084, *p* = 0.0116; t = 3.961, *p* = 0.0027; Figures 2C, F, *p* < 0.05).

### Effects of microglial depletion by PLX5622 on the expression of synaptic proteins, BDNF and sigma-1 receptor in the PFC of YL-0919-treated mice

**FIGURE 3:**
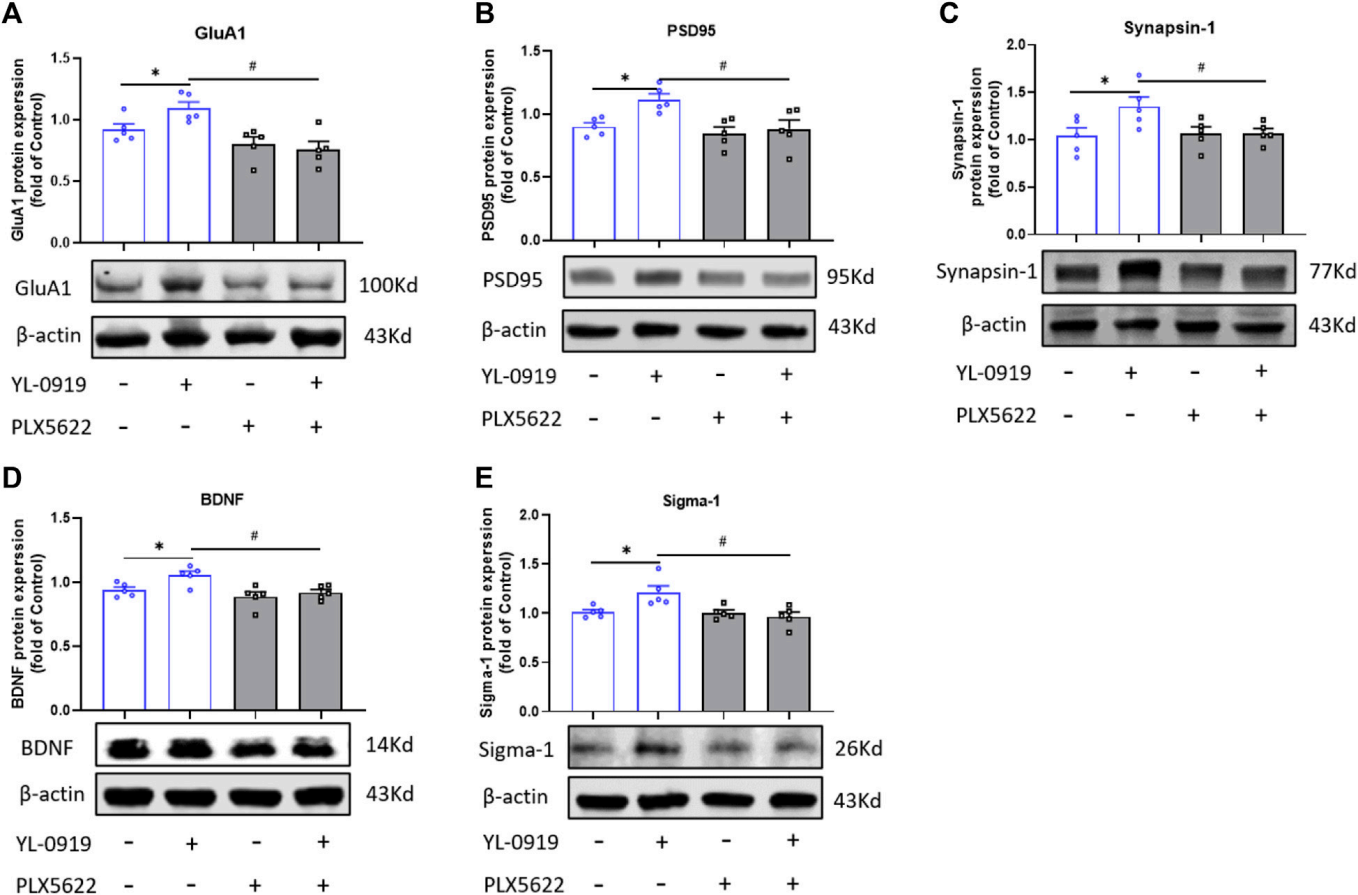
Effect of PLX5622 on expression of synaptic proteins, BDNF and sigma-1 receptor in the PFC of YL-0919-treated mice. Effect of PLX5622 on YL-0919-induced upregulation of GluA1 **(A)**, PSD-95 **(B)**, Synapsin-1 **(C)**, BDNF **(D)** and Sigma-1 **(E)** expression in the PFC of mice. veh + control vs. veh + YL-0919, *p < 0.05; veh + YL-0919 vs. PLX5622+YL-0919, ^#^ *p* < 0.05, Means ± SEM; *n* = 5.

To investigate the possible microglial mechanism of the rapid antidepressant action of YL-0919, the expression of synaptic proteins (GluA1, PSD-95, synapsin-1), BDNF, and sigma-1 receptor in the PFC were assayed. YL-0919 (2.5 mg/kg, i.g., administration for 9 consecutive days) significantly increased the expression of synaptic proteins (GluA1, PSD-95, synapsin-1) and BDNF in the PFC (t = 2.536, *p* = 0.0349; t = 3.949, *p* = 0.0042; t = 2.398, *p* = 0.0433; t = 2.968, *p* = 0.0179; t = 2.839, *p* = 0.0218; Figures 3A–D, *p* < 0.05). Compared with the YL-0919-treated group, the upregulation of GluA1, PSD-95, Synapsin-1, and BDNF by YL-0919 were significantly blocked by PLX5622 (t = 4.123, *p* = 0.0033; t = 2.776, *p* = 0.0241; t = 2.565, *p* = 0.0344; t = 3.460, *p* = 0.0086; Figures 3A–D, *p* < 0.05). Mounting studies suggested a crucial role of BDNF in rapid antidepressant action (Song et al., 2017). Sigma-1 receptor was expressed on neuron as well as microglia, and participated in BDNF modulation (Mysona et al., 2018; Wang et al., 2022). Sigma-1 receptor was also verified to be an important target of YL-0919 (Ren et al., 2023). YL-0919 enhanced the protein expression of Sigma-1 receptor (t = 2.839, *p* = 0.0218; Figure 3E, *p* < 0.05), which was blocked by PLX5622 (t = 3.060, *p* = 0.0156; Figure 3E, *p* < 0.05). These results suggested that YL-0919 promoted the expression of synaptic proteins and BDNF with the rapid antidepressant action simultaneously, and microglia were significantly involved in the increased expression of synaptic plasticity, BDNF and sigma-1 receptors by YL-0919 treatment.

## Discussion

In this study, we identified that depletion of microglia blocked the rapid antidepressant effects of ketamine and YL-0919, and also restrained the YL-0919-induced upregulation on synaptic proteins and BDNF in the PFC. Microglia played a vital role in the fast antidepressant procedures of ketamine and YL-0919, and were imperative to the rapid regulation of synaptic plasticity and BDNF by YL-0919.

In animal models, YL-0919 (1.25–2.5 mg/kg, i.g.) produced a rapid acting antidepressant effect in chronic unpredictable stressed (CUS) rodents within 3–5 days (Ran et al., 2018; Sun et al., 2019). In addition, YL-0919 also significantly improved depressive-like behaviors in cynomolgus monkeys subjected to CUS within 9d (Yin et al., 2020). The 2.5 mg/kg dose of YL-0919 (i.g.) from previous experiments was also selected in the present study. However, fluoxetine, the traditional first-line SSRI antidepressant, required 20–22 consecutive days to produce similar antidepressant behavior activity in the CUS rats and monkeys (Ran et al., 2018; Yin et al., 2020). Consistent with the different onset time in CUS model, our previous studies demonstrated that immobility duration in the TST and FST could be reduced 24 h after the last administration of YL-0919 (administration for 5–7 consecutive days) in the normal mice (Zhang et al., 2022). Whereas, the similar antidepressant effect was performed 24 h after the last administration of fluoxetine (administration for 14–21 consecutive days) in the normal mice (Yin et al., 2021; Zhang et al., 2022). The scheme of drug administration could be applied to verify the rapid onset time of antidepressants in the model of normal mice, which was adopted in this study to validate the role of microglia in the fast antidepressant action of ketamine and YL-0919.

Microglia are among the most potent modulators of neuronal activity and CNS homeostasis (Jurga et al., 2020; Troubat et al., 2021; Yin et al., 2021). Adjustment of microglial function is considered as a potential strategy for the depression treatment (Farhangian et al., 2023), and may also contribute to action of antidepressants. This study found that microglial depletion by PLX5622 reversed the rapid antidepressant activity of ketamine in normal mice. The data were consistent with the very limited report demonstrating that PLX3397 blocked the antidepressant effect of (*R*)-ketamine in CSDS susceptible mice (Yao et al., 2022), suggesting that microglia were involved in the rapid onset of ketamine. Some studies indicated that the activation of CSF-1R and TGF-β1 signaling were crucial for maintaining microglial survival (Zhang et al., 2020; Zhang et al., 2022). TGF-β1 could enhance synaptic plasticity by up-regulating the expression of BDNF and Trkb, which was a vital factor for the antidepressant action of (*R*)-ketamine (Loftis et al., 2010; Hashimoto, 2020).

Previous studies showed that YL-0919 treatment for 5 days significantly activated the BDNF-mTOR pathway and increased the synthesis of synaptic proteins and BDNF underlying enhanced dendritic complexity in the PFC (Li, 2020). The activation of the BDNF-mTOR pathway and regulation of synaptic plasticity mediated the rapid action of YL-0919. Similarly, studies confirmed that BDNF played an essential role in the rapid antidepressant of ketamine (Autry et al., 2011). Enhancement in synaptic plasticity and BDNF expression might predict a unique mechanism for fast-acting anti-depression. More importantly, depletion of microglia blocked the up-regulating effect of YL-0919 on synaptic protein and BDNF expression in the PFC. Our results suggested that microglia might be involved in the rapid regulation of synaptic plasticity and BDNF by YL-0919, which might be the vital point in the rapid onset of YL-0919.

Microglia are the only type of cells that express CSF-1R in the CNS, and CSF-1R inhibitor PLX5622 can deplete specifically cerebral microglia (Elmore et al., 2014; Tang et al., 2018; Liang et al., 2019). In this study, PLX5622 diet depleted 92% of microglia in the prefrontal cortex after consecutive administration for 19d, consistent with previous reports that the number of microglia were reduced by 90% with 2 weeks of PLX5622 administration (Elmore et al., 2014). In accordance with the previous experiments (Elmore et al., 2014; Worthen et al., 2020), our study indicated that microglial depletion by PLX5622 did not induce depressive-like behaviors in normal mice. However, PLX5622 could reverse the rapid antidepressant activity of ketamine and YL-0919, indicating the essential role of microglia in the rapid onset of ketamine and YL-0919. The immunofluorescence results demonstrated that PLX5622 administration for 19 days greatly depleted microglia in the PFC, and YL-0919/ketamine could promote the proliferation of the remaining microglia. Ketamine and YL-0919 regulated microglia proliferation in the microglial depletion model, suggesting that microglial function, instead of the number of microglia might adjust the fast antidepressant effect of ketamine and YL-0919. More experiments need to be further verified.

In addition, our recent studies demonstrated that YL-0919 was a high-affinity agonist of the sigma-1 receptor (Ren et al., 2023). Activation of Sigma-1 receptors promoted nerve regeneration and BDNF secretion (Hashimoto, 2013; Ji et al., 2017), and Sigma-1 receptors were also expressed on microglia (J. Jia et al., 2018). Our study found that YL-0919 increased the expression of sigma-1 in the PFC, and the crucial role of microglial sigma-1 receptors in the rapid antidepressant of YL-0919 and regulation of microglia function were worth in-depth study.

In conclusion, the current data revealed that microglia contributed to the rapid antidepressant of ketamine and YL-0919, and microglia participated in the upregulation of synaptic plasticity by YL-0919. Therefore, it is likely that microglial regulation would be a new direction for fast onset of anti-depression.

## Data Availability

The original contributions presented in the study are included in the article/supplementary material, further inquiries can be directed to the corresponding authors.
