# Corrigendum: Essential role of microglia in the fast antidepressant action of ketamine and hypidone hydrochloride (YL-0919)

**DOI:** 10.3389/fphar.2023.1232874

**Published:** 2023-06-14

**Authors:** Hai-Xia Chang, Wei Dai, Jin-Hao Bao, Jin-Feng Li, Ji-Guo Zhang, Yun-Feng Li

**Affiliations:** ^1^ College of Pharmacy, Shandong First Medical University and Shandong Academy of Medical Sciences, Taian, China; ^2^ Beijing Institute of Basic Medical Sciences, Beijing, China; ^3^ State Key Laboratory of Toxicology and Medical Countermeasures, Beijing Key Laboratories of Neuropsychopharmacology, Institute of Pharmacology and Toxicology, Beijing, China

**Keywords:** microglia, fast antidepressant action, ketamine, YL-0919, synaptic proteins

In the published article, there was an error in Figure 1I as published. The SEM bars of Figure 1I were not shown, and Figure 1I was revised. The corrected Figure 1 and its caption appear below.

**FIGURE 1:**
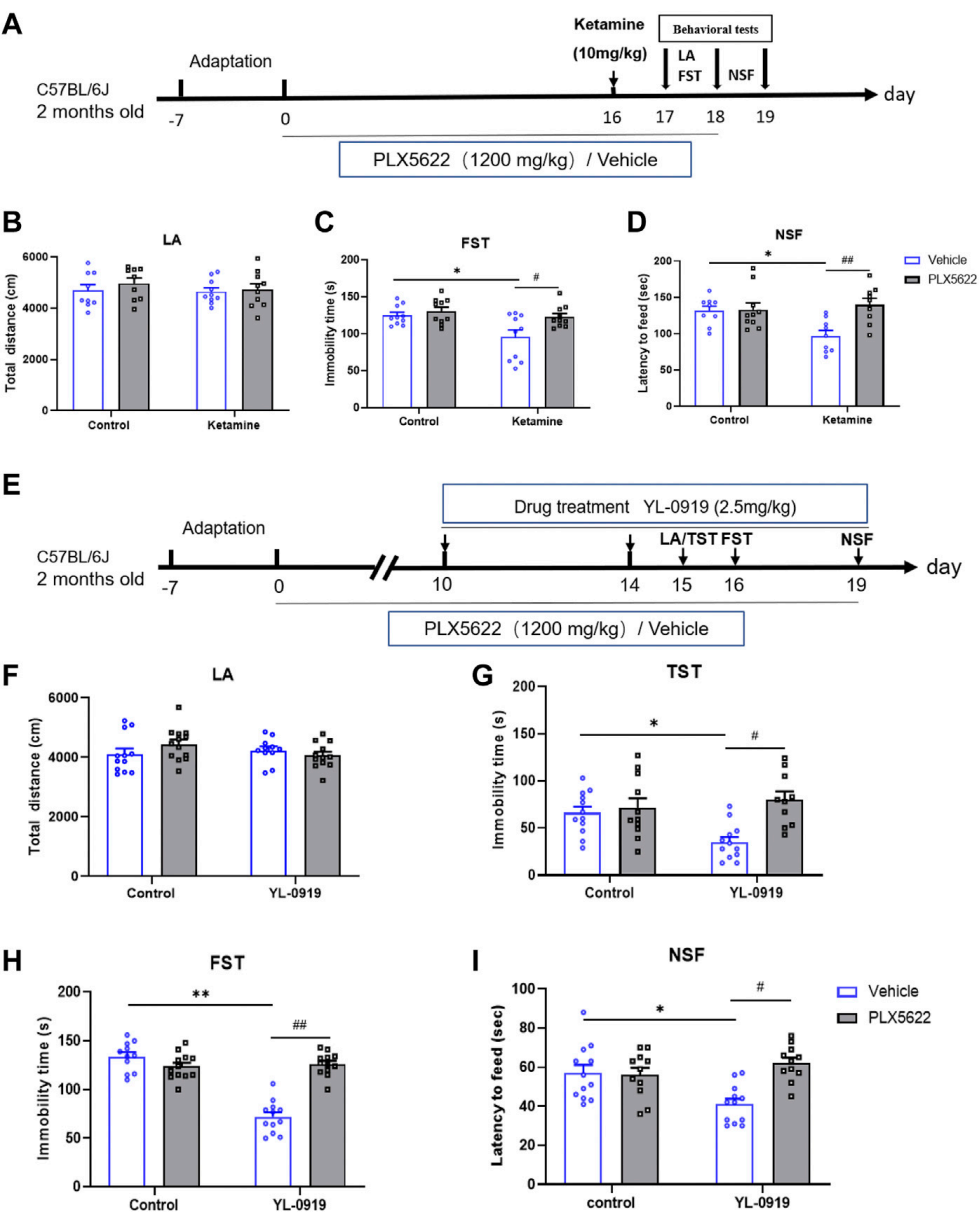
Schematic diagram illustrating the effects of PLX5622 on rapid antidepressant action of ketamine **(A)**; Effects of PLX5622 on behavioral tests including locomotor activity **(B)**, immobility duration in FST **(C)** and latency to feed **(D)** in ketamine-treated mice. veh + control vs. veh + ketamine, *p < 0.05; veh + ketamine vs. PLX5622+ketamine, ^#^ *p* < 0.05, ^##^ *p* < 0.01, *n* = 9–10. Schematic diagram illustrating the effects of PLX5622 on rapid antidepressant action of YL-0919 **(E)**; Effects of PLX5622 on behavioral tests including locomotor activity **(F)**, immobility duration in TST **(G)** and FST **(H)**; and latency to feed **(I)** in YL-0919-treated mice, veh + control vs. veh + YL-0919, *p < 0.05, **p < 0.01; veh + YL-0919 vs. PLX5622+YL-0919, ^#^ *p* < 0.05, ^##^ *p* < 0.01; Means ± SEM, *n* = 11–12.

In the published article, there was an error. The related description needs to be adjusted.

A correction has been made to **Abstract**, “*Results*,” paragraph number 01.

This sentence previously stated:

“In addition, the immobility time in TST and FST as well as latency to feed in NSFT were reduced 24 h after the intragastric (i.g.) administration of YL-0919 (2.5 mg/kg), and the rapid antidepressant effect of YL-0919 was also blocked by the microglial depletion using PLX5622.”

The corrected sentence appears below:

“In addition, the immobility time in TST and FST as well as latency to feed in NSFT were reduced 24 h after the intragastric (i.g.) administration of YL-0919 (2.5 mg/kg, administered for 5–6 consecutive days), and the rapid antidepressant effect of YL-0919 was also blocked by the microglial depletion using PLX5622.”

A correction has been made to **Introduction**, paragraph number 04.

This sentence previously stated:

“YL-0919, a novel antidepressant compound independently developed by our institute, was tested to be a sigma-1 receptor agonist (Ren et al., 2023).”

The corrected sentence appears below:

“YL-0919, a novel antidepressant compound developed by our institute, was tested to be a sigma-1 receptor agonist (**Ren et al., 2023**).”

A correction has been made to **Introduction**, paragraph number 04.

This sentence previously stated:

“In the clinical trial (phase IIa) with 45 depressive patients, YL-0919 showed a fast antidepressant response within 1 week.”

This sentence can be deleted.

A correction has been made to **Discussion**, paragraph number 02.

This sentence previously stated:

“In animal models, YL-0919 (1.25–2.5 mg/kg, i.g.) produced a rapid acting antidepressant effect in chronic unpredictable stressed (CUS) rodents within 3–5 days (Chen et al., 2018; Ran et al., 2018).”

The corrected sentence appears below:

“In animal models, YL-0919 (1.25–2.5 mg/kg, i.g.) produced a rapid acting antidepressant effect in chronic unpredictable stressed (CUS) rodents within 3–5 days (**Ran et al., 2018**; **Sun et al., 2019**).”

The authors apologize for these errors and state that this does not change the scientific conclusions of the article in any way. The original article has been updated.

